# Genome-Wide Association Study of Down Syndrome-Associated Atrioventricular Septal Defects

**DOI:** 10.1534/g3.115.019943

**Published:** 2015-07-20

**Authors:** Dhanya Ramachandran, Zhen Zeng, Adam E. Locke, Jennifer G. Mulle, Lora J.H. Bean, Tracie C. Rosser, Kenneth J. Dooley, Clifford L. Cua, George T. Capone, Roger H. Reeves, Cheryl L. Maslen, David J. Cutler, Eleanor Feingold, Stephanie L. Sherman, Michael E. Zwick

**Affiliations:** * Department of Human Genetics, Emory University, Atlanta, Georgia, 30033; † Department of Biostatistics, University of Pittsburgh, Pennsylvania 15261; ‡ Genetics and Molecular Biology Graduate Program, Graduate Division of Biological and Biomedical Sciences, Laney Graduate School, Atlanta, Georgia 30033; § Department of Epidemiology, Rollins School of Public Health, Atlanta, Georgia 30033; ** Department of Pediatrics, Sibley Heart Center Cardiology, Children’s Healthcare of Atlanta, Atlanta, Georgia 30033; †† Heart Center, Nationwide Children’s Hospital, Columbus, Ohio 43205; ‡‡ Kennedy Krieger Institute, Baltimore, Maryland 21205; §§ Department of Physiology and McKusick Nathans Institute for Genetic Medicine, School of Medicine, Johns Hopkins University, Baltimore, Maryland 21205; ††† Department of Human Genetics, Graduate School of Public Health, University of Pittsburgh, Pennsylvania 15261; *** Knight Cardiovascular Institute, and Department of Molecular and Medical Genetics, Oregon Health & Science University, Portland, Oregon 97239

**Keywords:** congenital heart defect, trisomy, birth defect, complex trait, aneuploidy

## Abstract

The goal of this study was to identify the contribution of common genetic variants to Down syndrome−associated atrioventricular septal defect, a severe heart abnormality. Compared with the euploid population, infants with Down syndrome, or trisomy 21, have a 2000-fold increased risk of presenting with atrioventricular septal defects. The cause of this increased risk remains elusive. Here we present data from the largest heart study conducted to date on a trisomic background by using a carefully characterized collection of individuals from extreme ends of the phenotypic spectrum. We performed a genome-wide association study using logistic regression analysis on 452 individuals with Down syndrome, consisting of 210 cases with complete atrioventricular septal defects and 242 controls with structurally normal hearts. No individual variant achieved genome-wide significance. We identified four disomic regions (1p36.3, 5p15.31, 8q22.3, and 17q22) and two trisomic regions on chromosome 21 (around *PDXK* and *KCNJ6* genes) that merit further investigation in large replication studies. Our data show that a few common genetic variants of large effect size (odds ratio >2.0) do not account for the elevated risk of Down syndrome−associated atrioventricular septal defects. Instead, multiple variants of low-to-moderate effect sizes may contribute to this elevated risk, highlighting the complex genetic architecture of atrioventricular septal defects even in the highly susceptible Down syndrome population.

Congenital heart defects (CHDs) comprise the most common birth defect and the largest contributor to infant mortality and morbidity (Moller *et al.* 1993; Boneva *et al.* 2001; Hoffman and Kaplan 2002; Cleves *et al.* 2003; Reller *et al.* 2008; Hoffman 2013; Shuler *et al.* 2013). CHDs represent a diverse group of structural and functional abnormalities of the heart that occur during early embryogenesis. With an incidence of nearly 1%, CHDs pose a serious global health concern and cause significant financial and social burden (Waitzman *et al.* 1994; Van Rijen *et al.* 2005; Russo and Elixhauser 2007) which remains despite major advances made to improve diagnoses and treatment (Fahed *et al.* 2013).

Many studies have shown that CHDs are heritable (Dennis and Warren 1981; Emanuel *et al.* 1983; Cripe *et al.* 2004; Lewin *et al.* 2004). Genetic studies using family-based linkage (Schott *et al.* 1998; Garg *et al.* 2003; French *et al.* 2012; Flaquer *et al.* 2013), genome-wide single-nucleotide polymorphism (SNP) or copy number variant (CNV) association (Greenway *et al.* 2009; Soemedi *et al.* 2012; Cordell *et al.* 2013a; Cordell *et al.* 2013b; Sailani *et al.* 2013; Ramachandran *et al.* 2014), and candidate gene/whole-exome sequencing (Robinson *et al.* 2003; Pierpont *et al.* 2007; Ackerman *et al.* 2012; Zaidi *et al.* 2013; Al Turki *et al.* 2014) have revealed genetic variants associated with CHD. As expected for a complex trait, the etiology of CHD also is found to be influenced by epigenetic and environmental exposures (Bean *et al.* 2011; Lage *et al.* 2012; Vallaster *et al.* 2012; Fung *et al.* 2013; Martinez *et al.* 2015).

Trisomy 21, the cause of Down syndrome (DS), has a birth prevalence of one in 700 and is the most common chromosomal aneuploidy that survives to term. Nearly 50% of newborns with DS have some form of CHD (Freeman *et al.* 1998, 2008). One of the common types of CHD associated with DS is atrioventricular septal defect (AVSD) or atrioventricular canal defect, a severe structural anomaly that requires surgery at a very young age. With a birth prevalence of 0.83 in 10,000 live births, AVSD is rare in the general population (Hartman *et al.* 2011); however, in the trisomy 21 population, the associated risk is increased by 2,000-fold, occurring in about 20% of individuals with DS having AVSD (Freeman *et al.* 2008). Among all AVSD cases, more than 65% occur in children with DS (Ferencz *et al.* 1989). Yet despite the dramatically increased risk of AVSD among children with DS, 80% of infants with DS do not have AVSD, and nearly half of them do not have any CHD.

Together, these epidemiologic observations suggest that although trisomy 21 predisposes the heart to form abnormally, genetic variants on chromosome 21 (ch21) or other chromosomes may act to modify the risk of developing an AVSD. Thus, individuals with DS or trisomy 21 can be considered a “sensitized” population with respect to CHD. The study of this cohort may help reveal CHD susceptibility factors, much as has been done in model organisms (Zwick *et al.* 1999; St Johnston 2002; Li *et al.* 2012).

Our original hypothesis was that the genetic architecture of this increased risk may be relatively simple. A few common modifying variants could have a large effect size on CHD in a trisomic background while having little or no effect in a euploid individual. This hypothesis was demonstrated in a recent study using a mouse model of DS, in which the authors showed that mutations in AVSD risk factor genes *Creld1* and *Hey2* were individually benign on a euploid background but substantially increased risk for septal defects when expressed on a trisomic background or when inherited together in euploid mice (Li *et al.* 2012). If our hypothesis is correct, these common variants would be expected to produce a stronger statistical signal in a genome-wide association study among individuals chosen from the extreme ends of the phenotypic distribution. Here we report the results of a genome-wide association study comparing individuals with DS and complete AVSD (DS + AVSD, cases = 210) with individuals with DS and a structurally normal heart (DS + NH, controls = 242).

## Materials and Methods

### Study subjects

The study sample described is the same as that used in Ramachandran *et al.* (2014) to investigate the role of CNVs in DS-associated AVSD. Details regarding the recruitment and enrollment methods have been documented previously (Freeman *et al.* 2008; Locke *et al.* 2010). Briefly, participants with a diagnosis of full trisomy 21 were enrolled through multiple centers across the United States. Protocols were approved by institutional review boards at each participating center. Written and oral consent were obtained from custodial parents for each participant because most of the subjects themselves were unable to give consent as the result of cognitive deficits. Cases were defined as individuals with DS who had a complete, balanced AVSD documented most often by echocardiogram or surgical reports (DS + AVSD). Control subjects were defined as those with a structurally normal heart (DS + NH), documented by echocardiogram in the vast majority. Individuals with patent foramen ovale or patent ductus arteriosus were included in the control population, because these defects affect structures with different ontology. Only participants whose mother reported being non-Hispanic European Americans were included in the current study.

### Genotyping

Genomic DNA was isolated from low passage lymphoblastoid cell lines (between one and four passages) and genotyping was carried out using the Affymetrix Genome-Wide Human SNP 6.0 array at Emory University according to manufacturer’s instructions. Genotype calling was performed using the Birdseed algorithm (version 2), as implemented in the Affymetrix Power Tools software (APT 1.12.0). To assess initial quality of arrays, we followed Affymetrix’s recommended quality control thresholds: Individual arrays with <86% call rate, <0.04 contrast quality control (QC), and mismatched gender concordance were excluded from downstream analyses. These thresholds were selected because genotype calling of SNPs on the trisomic ch21 using standard methods (APT 1.12.0) is unreliable and lowers the overall call rate. Genotype calling for ch21 in trisomic individuals was performed at the University of Pittsburgh using methods similar to those described in Lin *et al.* (2008).

### Quality control steps and statistical analyses

Before conducting association tests, we performed rigorous sample and SNP cleaning. The details of this process are documented in Ramachandran *et al.* (2014). In brief, QC was done on 471 samples. Nine samples were excluded because of poor data completeness or inconsistent family structure. An additional seven were excluded as outliers defined by principal component analysis performed using Eigenstrat software v4 (Price *et al.* 2006). The plot of principal component analysis for the trisomic sample set in the final analyses is provided in Supporting Information, Figure S1 (Ramachandran *et al.* 2014). The final sample set consisted of a total of 210 DS + AVSD cases and 242 DS + NH controls. Data cleaning was performed in PLINK v1.07 for the non-ch21 autosomal SNPs (855,628 SNPs) (Purcell *et al.* 2007). SNPs with >5% missing data, minor allele frequency <5%, as well as those that deviated from Hardy-Weinberg equilibrium (*P* < 1*10^−5^) were excluded from analysis. A total of 606,195 autosomal SNPs were retained for downstream genome-wide association analysis, whereas 249,433 SNPs were excluded.

We performed the case-control association test using logistic regression analysis in an additive model adjusting for the top five eigenvectors as covariates, implemented in PLINK v1.07 with a 95% confidence interval for odds ratio (OR). For SNPs on ch21, the association test was conducted in a similar manner (additive model, adjusting for top five covariates) using an in-house R script to account for the four genotype calls per SNP as expected with trisomy. Datagraph 3.2 was used to generate Manhattan plots (http://www.visualdatatools.com/DataGraph/). Locus plot showing the recombination rate and -log_10_ *P* value of SNPs at the candidate regions was made using LocusZoom (Pruim *et al.* 2010). The regulatory activity of the candidate regions was visualized through multiple annotation tracks using the University of California, Santa Cruz genome browser (Rosenbloom *et al.* 2013).

We assessed the power of our study using an additive disease model with a disease prevalence of 20% in the DS population. We assumed an underlying quantitative liability trait, with disease (AVSD) being present when exceeding a threshold on the liability scale. Minor allele frequencies were allow to vary from ∼0.1 to 0.5, assuming 210 cases and 242 controls. Alpha was set at 0.05 / total number of markers (606,195, *i.e.*, Bonferroni correction was for the total number of autosomal markers excluding those on ch21).

### Data availability

Raw genotype data are available in Gene Expression Omnibus (GEO) data respository with accession number: GSE60607.

## Results

### Association of autosomal (non-ch21) SNPs with AVSD

**Figure 1:**
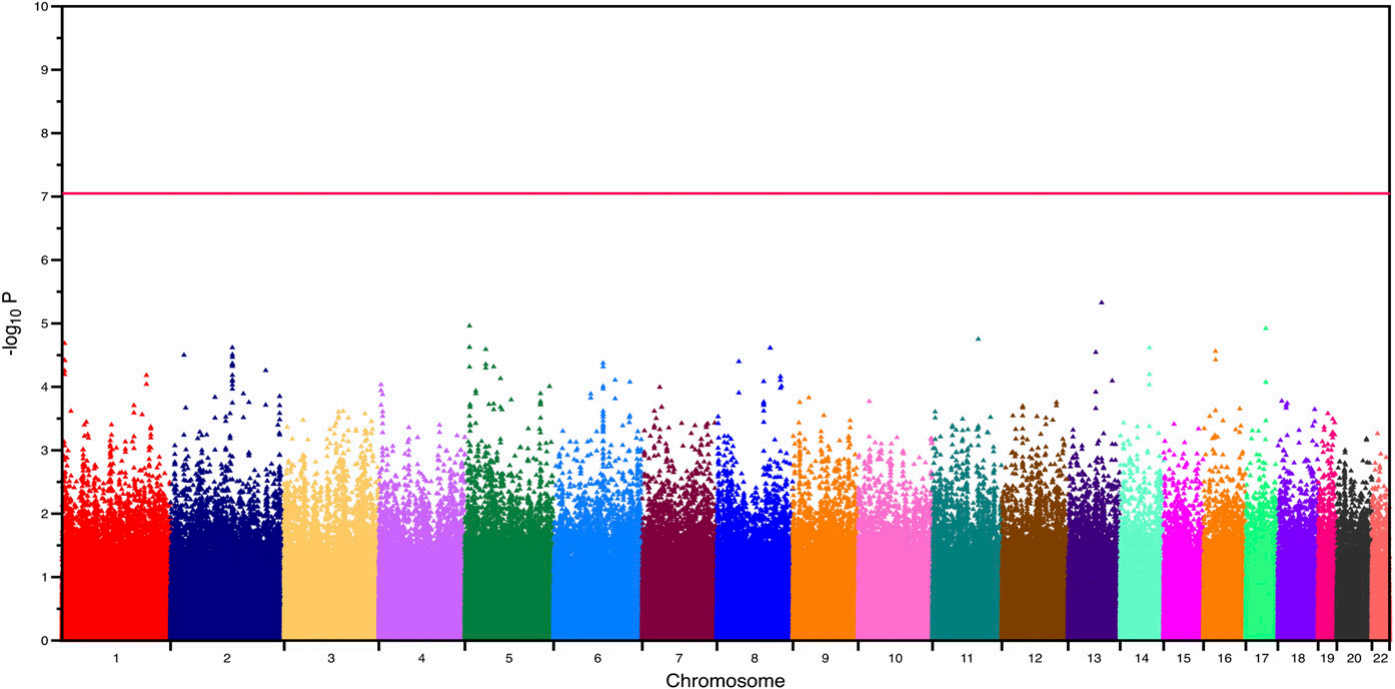
Manhattan plot of the genome-wide association analysis based on the case-control dataset for the non-chromosome 21 autosomal single-nucleotide polymorphisms. The horizontal red line denotes the *P*-value threshold for genome-wide significance.

We first sought to identify common autosomal (non-ch21) SNP variants associated with an increased risk of DS-associated AVSD. We genotyped 452 trisomic individuals, consisting of 210 cases and 242 controls of European Americans ancestry. A complete list of SNPs with *P*-values < 1*10^−4^ are provided in Table S1. The quantile-quantile plot showed good agreement between the observed and expected *P*-values (Figure S2). We did not find any SNPs that exceeded genome-wide significance (*P* < 8*10^−8^, Figure 1). We had 80% power to detect a common marker that explained 10–15% of variance or an OR greater than 2.0 after Bonferroni correction. We thus conclude there are no common alleles with large effect size (OR > 2.0) that explain the increased risk of DS-associated AVSD.

**Figure 2:**
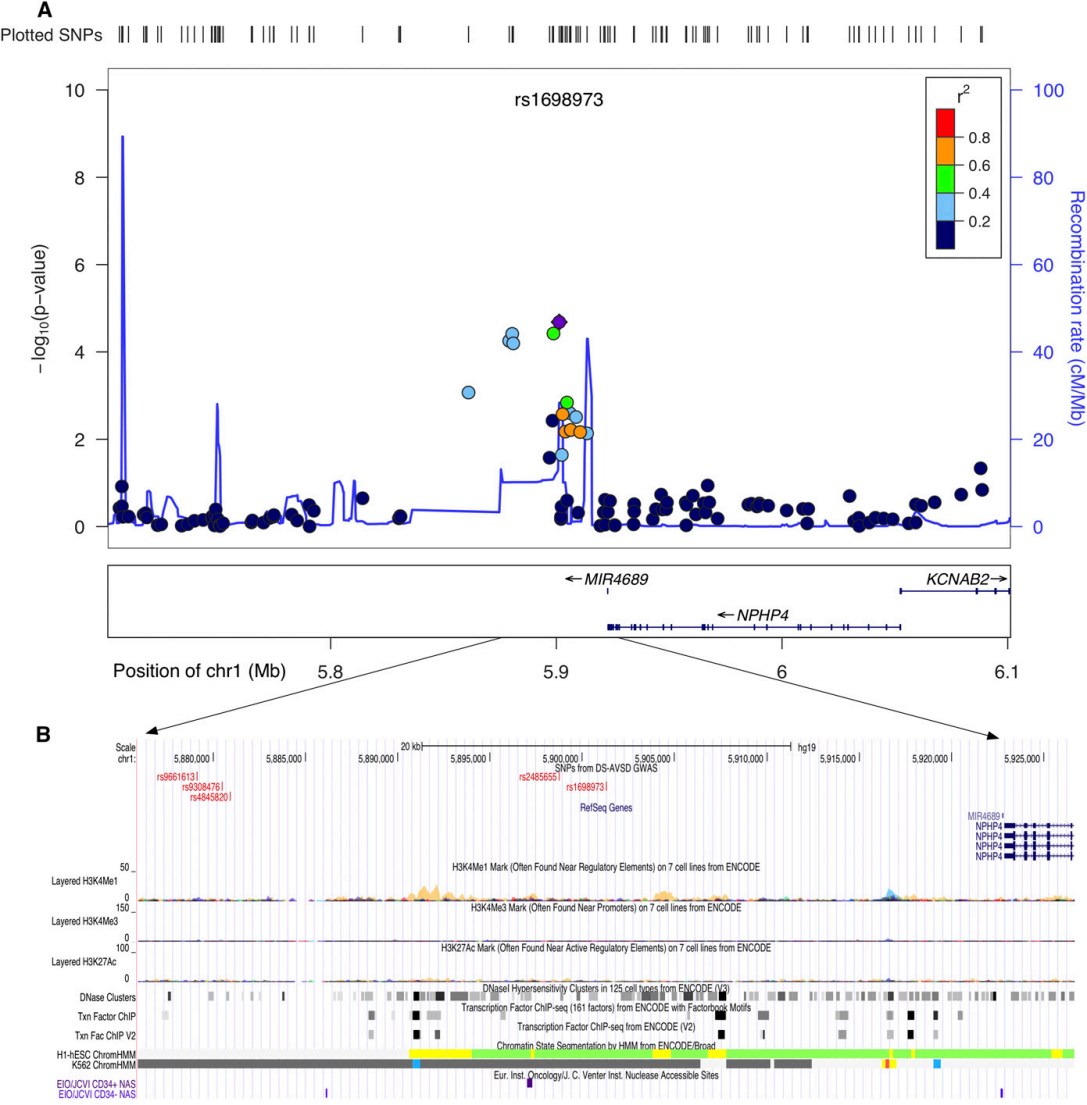
Genomic region at 1p36.3 with suggestive evidence of association with Down syndrome−associated atrioventricular septal defect. (A) and (B) show the locus zoom plot and University of California, Santa Cruz (UCSC) image at the corresponding genomic region (human genome build 19, hg19). (A) Locus zoom plot of the region of association at single-nucleotide polymorphism (SNP) with strongest signal (rs1698973). Linkage disequilibrium (LD) between this SNP (purple diamond) and nearby markers is color-coded based on the strength of the LD. The left Y-axis shows the -log_10_ of the association *P*-value and the right Y-axis indicates the recombination rate across each region. The position on the chromosome (hg19) and the nearby genes are shown below the X-axis. (B) UCSC browser image shows evidence of regulatory activity. Included are a custom track showing the location of most significant SNPs and the annotation tracks from ENCODE showing regulatory activity.

**Figure 3:**
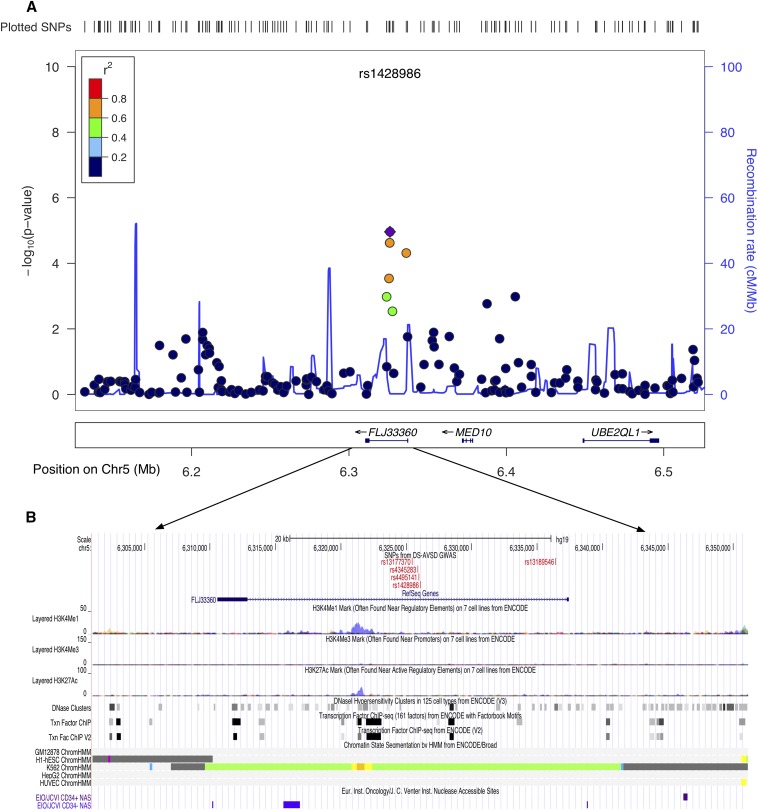
Genomic region at 5p15.3 with suggestive evidence of association with Down syndrome−associated atrioventricular septal defect. (A) Locus zoom plot of the region of association at single-nucleotide polymorphism (SNP) with strongest signal (rs1428986). Linkage disequilibrium (LD) between this SNP (purple diamond) and nearby markers is color-coded based on the strength of LD. The left Y-axis shows the -log_10_ of the association *P*-value and the right Y-axis indicates the recombination rate across each region. The position on the chromosome (hg19) and the nearby genes are shown below the X-axis. (B) University of California, Santa Cruz browser image shows evidence of regulatory activity. Included are a custom track showing the location of most significant SNPs and the annotation tracks from ENCODE showing regulatory activity.

We identified four regions with suggestive evidence of association at 1p36.3, 5p15.31, 8q22.3, and 17q22 (Table 1). A closer examination of the regions tagged by these SNPs revealed they were in close proximity to genes with roles important for heart development and/or function. Furthermore, multiple annotation tracks from ENCODE indicated strong regulatory activity, providing further evidence of putative function. Within the 1p36.3 candidate region, the strongest signal (rs1698973) was located adjacent to *NPHP4*, a ciliome gene (Figure 2) (Habbig *et al.* 2011). Interestingly, recent studies on heart phenotypes in a trisomic background implicate a significant role for ciliome genes in the etiology of DS-associated AVSD (Ripoll *et al.* 2012; Ramachandran *et al.* 2014; Li *et al.* 2015). The second region of interest was at 5p15.31. The strongest signal at this region (rs1428986, *P* < 1.09*10^−5^) falls within *FLJ33360*, a long noncoding RNA gene (lncRNA). lncRNAs are associated with gene regulation, and recent studies point to an emerging role in the pathophysiology of complex human diseases (Wapinski and Chang 2011; Ma *et al.* 2012). Adjacent to *FLJ33360* is the *MED10* gene (Figure 3). Mutations in *MED10* have been associated with cardiac defects (Lin *et al.* 2007). In addition, multiple ENCODE annotation tracks suggest a weak enhancer activity at both 1p36.3 and 5p15.31 regions (Figure 2 and Figure 3). The third candidate region, 8q22.3 (rs3107646 and rs1522707, both SNPs with *P* < 2.4*10^−5^), is located next to *FZD6*, encoding Wnt receptor protein. Wnt signaling plays a key role in cardiovascular physiology (reviewed by Cohen *et al.* 2008). Moreover, annotations from ENCODE indicate a strong enhancer activity at this region (Figure S3). The fourth region of interest, at 17q22 (rs7225274, *P* < 1.2*10^−5^), is located at an intergenic region. Evidence from multiple annotation tracks suggests a strong regulatory activity. This region includes several binding sites for transcription factors, including GATA proteins and NR2F2 (Figure S4). Mutations in both of these genes have been associated with CHD, including AVSD (Garg *et al.* 2003; Al Turki *et al.* 2014). Nevertheless, the association and ENCODE findings at our top four regions are not genome-wide statistically significant and require replication in an independent cohort.

**Table 1:** SNPs with strongest signals indicating suggestive association in candidate regions

Chr*^a^*	SNP	Base Position hg19	Gene*^b^*	GWAS *P*-Value	GWAS OR (95% CI)
1p36.3	rs1698973	5901288	(NPHP4)	2.068*10^−5^	0.46 (0.32−0.66)
5p15.31	rs1428986	6326059	FLJ33360	1.093*10^−5^	1.88 (1.42−2.49)
8q22.3	rs3107646	104285531	(FZD6)	2.436*10^−5^	0.40 (0.26−0.61)
8q22.3	rs1522707	104287845	(FZD6)	2.436*10^−5^	0.40 (0.26−0.61)
17q22	rs7225247	52933176	(TOM1L1)	1.199*10^−5^	1.86 (1.41−2.46)

SNP, single-nucleotide polymorphism; GWAS, genome-wide association study; OR, odds ratio; 95% CI, 95% confidence interval.

a Chromosome.

b For SNPs located within genes, gene names are listed; for intergenic SNPs, the nearest protein coding gene is listed in parenthesis.

### Association of ch21 SNPs with AVSD

**Figure 4:**
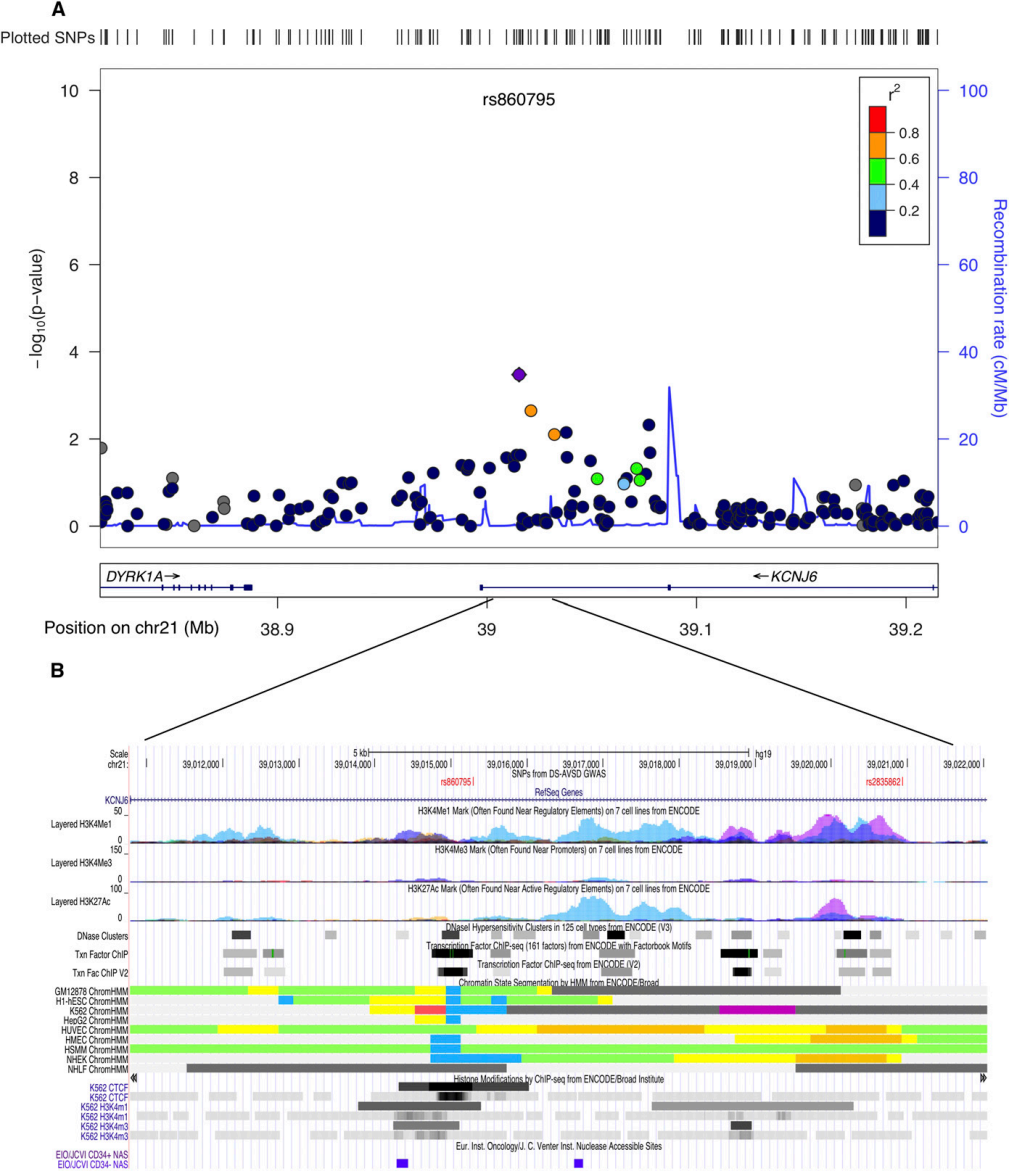
Genomic region on chromosome 21q22.1 at the *KCNJ6* gene showing suggestive evidence of association. (A) Locus zoom plot of the region of association at single-nucleotide polymorphism (SNP) with strongest signal on ch21 (rs860795). Linkage disequilibrium (LD) between this SNP (purple diamond) and nearby markers is color-coded based on the strength of LD. The left Y-axis shows the -log_10_ of the association *P*-value and the right Y-axis indicates the recombination rate across each region. The position on the chromosome (hg19) and the nearby genes are shown below the X-axis. (B) University of California, Santa Cruz browser image shows evidence of regulatory activity. Included are a custom track showing the location of most significant SNPs and the annotation tracks from ENCODE showing regulatory activity.

**Figure 5:**
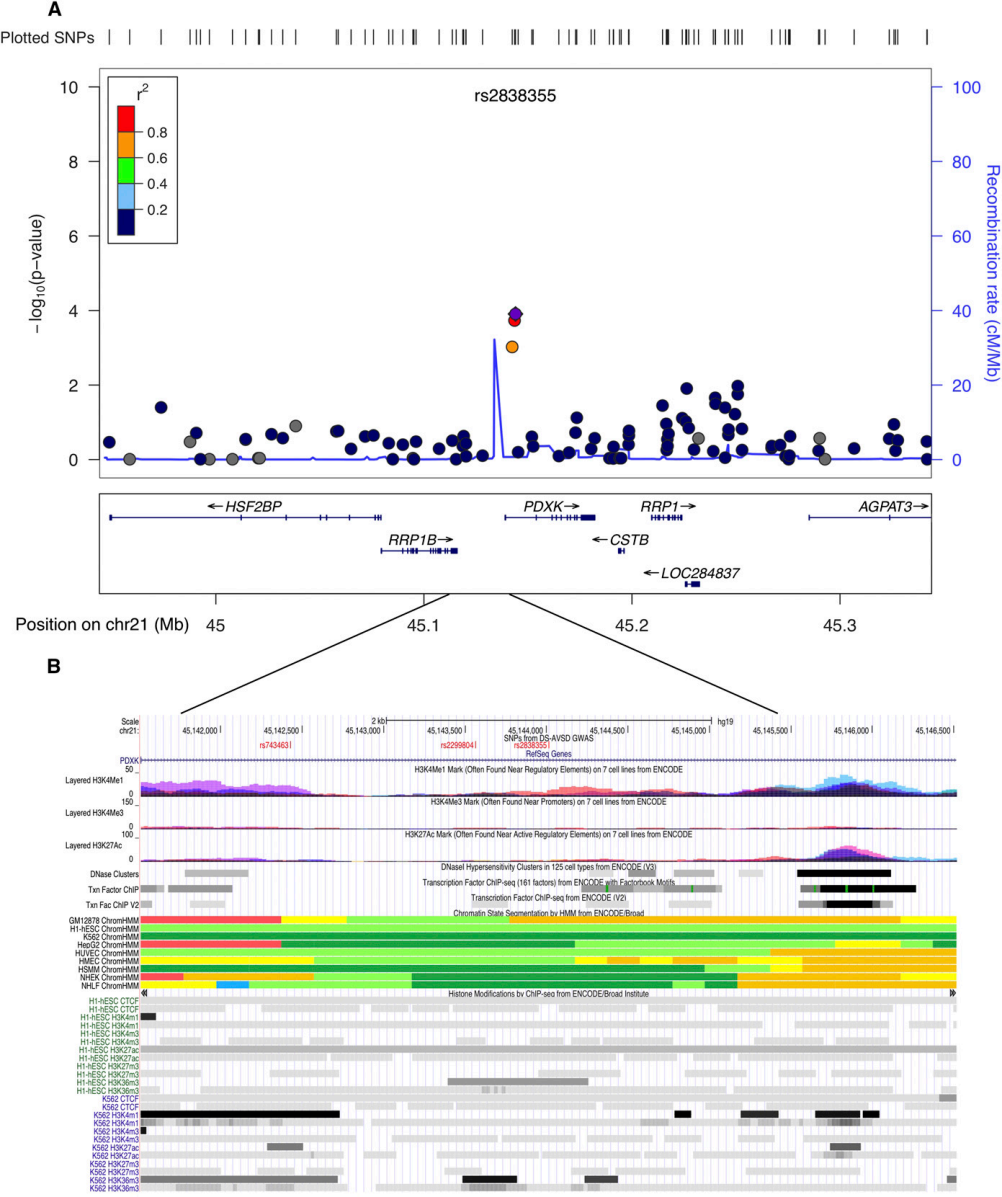
Genomic region on chromosome 21q22.3 at the *PDXK* gene showing suggestive evidence of association. (A) Locus zoom plot of the region of association at single-nucleotide polymorphism (SNP) with strongest signal on ch21 (rs2838355). Linkage disequilibrium (LD) between this SNP (purple diamond) and nearby markers is color-coded based on the strength of LD. The left Y-axis shows the -log_10_ of the association *P*-value and the right Y-axis indicates the recombination rate across each region. The position on the chromosome (hg19) and the nearby genes are shown below the X-axis. (B) University of California, Santa Cruz browser image shows evidence of regulatory activity. Included are a custom track showing the location of most significant SNPs and the annotation tracks from ENCODE showing regulatory activity.

A case-control association test was carried out separately for 12,584 SNPs on the trisomic ch21. None of the ch21 SNPs achieved genome-wide significance. However, two regions, *KCNJ6* at 21q22.13 and the *PDXK* gene at 21q22.3, were noteworthy. SNPs at these locations had the lowest *P*-values. The strongest signal at *KCNJ6* was rs860795 (*P* < 3.3*10^−4^) (Figure 4) and at *PDXK*, rs2838355, had a *P* < 1*10^−4^ (Figure 5). Overexpression of genes at these regions has been shown to be associated with DS pathology, including heart defects. Furthermore, evidence from multiple ENCODE annotation tracks indicate strong regulatory activity, possibly an enhancer/promoter function (Figure 4 and Figure 5). The list of SNPs on ch21 (*P* < 1*10^−3^) and the corresponding genomic location from the case-control association data are provided in the supplemental section (Table S2). Nevertheless, the association and ENCODE findings at the two ch21 are not genome-wide statistically significant and require replication in an independent cohort.

## Discussion

Infants with trisomy 21, or DS, are at increased risk for developing congenital heart defects, especially AVSD. Compared with the general euploid population, individuals with trisomy 21 have a 2000-fold increased risk of developing AVSD. We conducted a genome-wide association study to identify the genetic variants contributing to this phenotype using a carefully phenotyped collection of 210 DS + AVSD cases and 242 DS + NH controls to complement our genome-wide CNV study (Ramachandran *et al.* 2014). Our study had 80% power to detect common variants with an odds ratio greater than 2.0; none of the SNPs we tested exceeded this threshold. We therefore conclude that the enormous increased risk of AVSD in infants with DS is not caused by a few common variants of large effect, as we originally hypothesized.

We did, however, identify four non-ch21 autosomal regions worthy of replication in an independent sample set. In the first region at 1p36.3, the most significant SNP, rs1698973 (*P* < 2.07*10^−5^), is located about 21 kb downstream of *NPHP4*. NPHP4 is involved in renal tubular development and function. *NPHP4* is expressed in heart, kidney, skeletal muscle, and liver, and mutations in *NPHP4* have been associated with cardiac laterality defects (French *et al.* 2012). Interestingly, cardiac laterality defects and renal dysfunction are hall marks of defects in the cilia genes (Hou *et al.* 2002; Fliegauf *et al.* 2007). In addition to its role in left-right patterning, compelling evidence from recent studies using mouse models implicate a complex and critical role for the cilium in CHD pathogenesis in a much broader context (Friedland-Little *et al.* 2011; Li *et al.* 2015). A gene expression study using a smaller trisomic sample set by Ripoll *et al.* (Ripoll *et al.* 2012) showed enrichment for ciliome genes in their DS+AVSD subjects. A recent genome-wide CNV analysis performed by our group using the same cohort as presented in this GWAS analyses showing a suggestive enrichment for rare deletions in the ciliome genes further supports a significant role for cilia genes in normal heart development in humans (Ramachandran *et al.* 2014).

The second candidate was rs1428986 (*P* < 1.09*10^−5^) at 5p15.31. This variant is located within an RNA gene, *FLJ33360*, and the adjacent protein coding gene, *MED10*, is located 50 kb downstream. *FLJ33360* belongs to the lncRNA class. Functionally, lncRNAs are implicated in diverse aspects of gene expression and protein synthesis, including epigenetic regulation and direct transcriptional regulation (Wilusz *et al.* 2009; Ma *et al.* 2012). Recent studies have shown that the expression of lncRNA is tissue-specific and, furthermore, disruptions in lncRNA have been linked to the pathology of many human diseases, ranging from neurodegeneration to cancer (Wapinski and Chang 2011; Gutschner *et al.* 2013). *FLJ33360* is expressed in the heart, but more evidence is required to establish its role in the etiology of CHD. MED10 plays a major role in the transcriptional regulation of RNA polymerase II−dependent genes (Sato *et al.* 2003). Interestingly, using zebrafish mutants, Lin *et al.* (2007) have shown that depletion of MED10 causes specific defects in cardiac cushion formation, possibly through Wnt and nodal signaling.

The third region that warrants attention was on 8q22.3. SNPs with the strongest association are located at an intergenic region flanked by *BAALC*, 45.3 kb upstream, and *FZD6*, 22.8 kb downstream. BAALC is believed to play a synaptic role and is expressed by neural and hematopoietic cells (Tanner *et al.* 2001), whereas FZD6 (frizzled class receptor6) protein is detected in multiple tissues, including adult heart, brain, and placenta. Frizzled proteins act as a receptor for Wnt proteins and play a role in signal transduction via the Wnt/beta-catenin pathway (Umbhauer *et al.* 2000). Wnt signaling has a critical role in the development of the heart (Hurlstone *et al.* 2003; Alfieri *et al.* 2010; Marinou *et al.* 2012).

The fourth region of interest was at 17q22, with the strongest association signal at rs7225247 (*P* < 1.2*10^−5^). This variant is flanked by the gene *KIF2B*, 1.03 Mb upstream, and *TOM1L1*, 44.7 kb downstream. *KIF2B* is moderately expressed in heart, and its activity is critical for spindle assembly and chromosome movement, whereas *TOM1L1* is involved in signaling pathways (Puertollano 2005; Manning *et al.* 2007). A region of high regulatory activity is located around 5 kb downstream of this variant, including binding sites for transcription factors, including GATA1, GATA2, GATA3, and NR2F2. The association between GATA proteins and congenital heart defects is well documented (Garg *et al.* 2003; Raid *et al.* 2009; Wang *et al.* 2012; Zheng *et al.* 2012; Li *et al.* 2014; Shan *et al.* 2014). A recent study implicates an association between rare sequence variants in *NR2F2* and AVSD in nonsyndromic individuals (Al Turki *et al.* 2014). All four of the candidate regions showed the presence of histone markers, DNase hypersensitivity clusters, transcription factor binding sites, and nuclease accessible sites indicating regulatory activity. Chromatin segmentation status from ENCODE associates these regions with weak enhancer/promoter activity.

Although none of the variants on ch21 reached genome-wide significance, we identified two regions worthy of further investigation in replication studies. The first region on ch21 that showed a suggestive association was at 21q22.13, where the most significant SNP, rs860795, is located in an intron of the *KCNJ6* gene. *KCNJ6* encodes a G-protein activated inward rectifier potassium channel, expressed in fetal heart, and its overexpression causes altered heart rate (Lignon *et al.* 2008). Interestingly, functional analyses on trisomic (DS) mouse model (*Dp(16)4Yey/+*) have implicated a 3.7-Mb “critical region” flanked by the *Ifnar1-Kcnj6* region on mouse chromosome 16 (Mmu16) in DS-related heart defects (Liu *et al.* 2011; Liu *et al.* 2014). A recent genome-wide association study on conotruncal and related heart defects in a disomic population uncovered suggestive evidence for an intronic SNP rs2267386 (22q13.1) within *KCNJ4*, a paralog of the *KCNJ6* gene (Agopian *et al.* 2014). The second ch21 candidate region showing evidence of association was at 21q22.3. The most significant SNP in this region, rs2838355 (*P* < 1*10^−4^), was located at the intronic region of the *PDXK* gene. PDXK is involved with vitamin B6 phosphorylation and is expressed ubiquitously. Evidence of significant overexpression of *PDXK* and its neighboring gene, *RRP1B* (23 kb upstream), in trisomic subjects argues for a role of these genes in DS pathology. However, as yet there is no specific association to any heart phenotype (Salemi *et al.* 2012). Both the ch21 candidate regions appear to have high regulatory activity, with potential enhancer/promoter functions (ENCODE). The Affymetrix 6.0 genotyping microarrays did not have a sufficient marker density to replicate the recent findings of association reported by Sailani (Sailani *et al.* 2013).

Most of the GWAS studies on heart conducted to date employ a phenotypically heterogeneous sample set, thereby diluting the power to detect specific associations (Larson *et al.* 2007; Cordell *et al.* 2013b; Hu *et al.* 2013; Agopian *et al.* 2014; Xu *et al.* 2014). Here, using individuals with DS as a “sensitized” population, we conducted a genome-wide association study on a carefully phenotyped collection of individuals from extreme ends of the spectrum. Although our study represents the largest DS-associated study conducted so far using a homogeneous heart phenotype, we are still underpowered to detect variants of modest to low effect sizes. Replication studies with larger sample sizes are required to confirm the suggestive regions we have identified here. Finally, despite the 2000-fold risk observed, this elevated risk cannot be explained easily, but instead represents a complex interplay with the increased dosage of ch21. Given the complex and multifactorial nature of the disorder, the next feasible approach would be to use whole genome-sequencing to characterize the genetic variants, along with epigenetic interactions and environmental factors in a larger patient cohort.

## References

[bib1] Ackerman C. Locke A. E. Feingold E. Reshey B. Espana K., 2012 An excess of deleterious variants in VEGF-A pathway genes in Down-syndrome-associated atrioventricular septal defects. Am. J. Hum. Genet. 91: 646–659. 23040494 10.1016/j.ajhg.2012.08.017 PMC3484504

[bib2] Agopian A. J. Mitchell L. E. Glessner J. Bhalla A. D. Sewda A., 2014 Genome-wide association study of maternal and inherited loci for conotruncal heart defects. PLoS One 9: e96057. 24800985 10.1371/journal.pone.0096057 PMC4011736

[bib3] Alfieri C. M. Cheek J. Chakraborty S. Yutzey K. E., 2010 Wnt signaling in heart valve development and osteogenic gene induction. Dev. Biol. 338: 127–135. 19961844 10.1016/j.ydbio.2009.11.030 PMC2814915

[bib4] Al Turki S. Manickaraj A. K. Mercer C. L. Gerety S. S. Hitz M. P., 2014 Rare variants in NR2F2 cause congenital heart defects in humans. Am. J. Hum. Genet. 94: 574–585. 24702954 10.1016/j.ajhg.2014.03.007 PMC3980509

[bib5] Bean L. J. Allen E. G. Tinker S. W. Hollis N. D. Locke A. E., 2011 Lack of maternal folic acid supplementation is associated with heart defects in Down syndrome: a report from the National Down Syndrome Project. Birth Defects Research (Part A). Clin. Mol. Teratol. 91: 885–893. 10.1002/bdra.22848 PMC3233972 21987466

[bib6] Boneva R. S. Botto L. D. Moore C. A. Yang Q. Correa A., 2001 Mortality associated with congenital heart defects in the United States: trends and racial disparities, 1979–1997. Circulation 103: 2376–2381. 11352887 10.1161/01.cir.103.19.2376

[bib7] Cleves M. A. Ghaffar S. Zhao W. Mosley B. S. Hobbs C. A., 2003 First-year survival of infants born with congenital heart defects in Arkansas (1993–1998): a survival analysis using registry data. Birth Defects Research (Part A). Clin. Mol. Teratol. 67: 662–668. 10.1002/bdra.10119 14703791

[bib8] Cohen E. D. Tian Y. Morrisey E. E., 2008 Wnt signaling: an essential regulator of cardiovascular differentiation, morphogenesis and progenitor self-renewal. Development 135: 789–798. 18263841 10.1242/dev.016865

[bib9] Cordell H. J. Bentham J. Topf A. Zelenika D. Heath S., 2013 a Genome-wide association study of multiple congenital heart disease phenotypes identifies a susceptibility locus for atrial septal defect at chromosome 4p16. Nat. Genet. 45: 822–824. 23708191 10.1038/ng.2637 PMC3793630

[bib10] Cordell H. J. Topf A. Mamasoula C. Postma A. V. Bentham J., 2013 b Genome-wide association study identifies loci on 12q24 and 13q32 associated with tetralogy of Fallot. Hum. Mol. Genet. 22: 1473–1481. 23297363 10.1093/hmg/dds552 PMC3596849

[bib11] Cripe L. Andelfinger G. Martin L. J. Shooner K. Benson D. W., 2004 Bicuspid aortic valve is heritable. J. Am. Coll. Cardiol. 44: 138–143. 15234422 10.1016/j.jacc.2004.03.050

[bib12] Dennis N. R. Warren J., 1981 Risks to the offspring of patients with some common congenital heart defects. J. Med. Genet. 18: 8–16. 7253006 10.1136/jmg.18.1.8 PMC1048650

[bib13] Emanuel R. Somerville J. Inns A. Withers R., 1983 Evidence of congenital heart disease in the offspring of parents with atrioventricular defects. Br. Heart J. 49: 144–147. 6824534 10.1136/hrt.49.2.144 PMC481276

[bib14] Fahed A. C. Gelb B. D. Seidman J. G. Seidman C. E., 2013 Genetics of congenital heart disease: the glass half empty. Circ. Res. 112: 707–720. 23410880 10.1161/CIRCRESAHA.112.300853 PMC3827691

[bib15] Ferencz C. Neill C. A. Boughman J. A. Rubin J. D. Brenner J. I., 1989 Congenital cardiovascular malformations associated with chromosome abnormalities: an epidemiologic study. J. Pediatr. 114: 79–86. 2521249 10.1016/s0022-3476(89)80605-5

[bib16] Flaquer A. Baumbach C. Pinero E. Garcia Algas F. De La Fuente Sanchez M. A., 2013 Genome-wide linkage analysis of congenital heart defects using MOD score analysis identifies two novel loci. BMC Genet. 14: 44. 23705960 10.1186/1471-2156-14-44 PMC3664624

[bib17] Fliegauf M. Benzing T. Omran H., 2007 When cilia go bad: cilia defects and ciliopathies. Nat. Rev. Mol. Cell Biol. 8: 880–893. 17955020 10.1038/nrm2278

[bib18] Freeman S. B. Taft L. F. Dooley K. J. Allran K. Sherman S. L., 1998 Population-based study of congenital heart defects in Down syndrome. Am. J. Med. Genet. 80: 213–217. 9843040

[bib19] Freeman S. B. Bean L. H. Allen E. G. Tinker S. W. Locke A. E., 2008 Ethnicity, sex, and the incidence of congenital heart defects: a report from the National Down Syndrome Project. Genet. Med. 10: 173–180. 18344706 10.1097/GIM.0b013e3181634867

[bib20] French V. M. Van De Laar I. M. Wessels M. W. Rohe C. Roos-Hesselink J. W., 2012 NPHP4 variants are associated with pleiotropic heart malformations. Circ. Res. 110: 1564–1574. 22550138 10.1161/CIRCRESAHA.112.269795 PMC3916111

[bib21] Friedland-Little J. M. Hoffmann A. D. Ocbina P. J. Peterson M. A. Bosman J. D., 2011 A novel murine allele of Intraflagellar Transport Protein 172 causes a syndrome including VACTERL-like features with hydrocephalus. Hum. Mol. Genet. 20: 3725–3737. 21653639 10.1093/hmg/ddr241 PMC3168284

[bib22] Fung A. Manlhiot C. Naik S. Rosenberg H. Smythe J., 2013 Impact of prenatal risk factors on congenital heart disease in the current era. J. Am. Heart Assoc. 2: e000064. 23727699 10.1161/JAHA.113.000064 PMC3698764

[bib23] Garg V. Kathiriya I. S. Barnes R. Schluterman M. K. King I. N., 2003 GATA4 mutations cause human congenital heart defects and reveal an interaction with TBX5. Nature 424: 443–447. 12845333 10.1038/nature01827

[bib24] Greenway S. C. Pereira A. C. Lin J. C. Depalma S. R. Israel S. J., 2009 De novo copy number variants identify new genes and loci in isolated sporadic tetralogy of Fallot. Nat. Genet. 41: 931–935. 19597493 10.1038/ng.415 PMC2747103

[bib25] Gutschner T. Hammerle M. Eissmann M. Hsu J. Kim Y., 2013 The noncoding RNA MALAT1 is a critical regulator of the metastasis phenotype of lung cancer cells. Cancer Res. 73: 1180–1189. 23243023 10.1158/0008-5472.CAN-12-2850 PMC3589741

[bib26] Habbig S. Bartram M. P. Muller R. U. Schwarz R. Andriopoulos N., 2011 NPHP4, a cilia-associated protein, negatively regulates the Hippo pathway. J. Cell Biol. 193: 633–642. 21555462 10.1083/jcb.201009069 PMC3166863

[bib27] Hartman R. J. Rasmussen S. A. Botto L. D. Riehle-Colarusso T. Martin C. L., 2011 The contribution of chromosomal abnormalities to congenital heart defects: a population-based study. Pediatr. Cardiol. 32: 1147–1157. 21728077 10.1007/s00246-011-0034-5

[bib28] Hoffman J. I., 2013 The global burden of congenital heart disease. Cardiovasc. J. Afr. 24: 141–145. 24217047 10.5830/CVJA-2013-028 PMC3721933

[bib29] Hoffman J. I. Kaplan S., 2002 The incidence of congenital heart disease. J. Am. Coll. Cardiol. 39: 1890–1900. 12084585 10.1016/s0735-1097(02)01886-7

[bib30] Hou X. Mrug M. Yoder B. K. Lefkowitz E. J. Kremmidiotis G., 2002 Cystin, a novel cilia-associated protein, is disrupted in the cpk mouse model of polycystic kidney disease. J. Clin. Invest. 109: 533–540. 11854326 10.1172/JCI14099 PMC150876

[bib31] Hu Z. Shi Y. Mo X. Xu J. Zhao B., 2013 A genome-wide association study identifies two risk loci for congenital heart malformations in Han Chinese populations. Nat. Genet. 45: 818–821. 23708190 10.1038/ng.2636

[bib32] Hurlstone A. F. Haramis A. P. Wienholds E. Begthel H. Korving J., 2003 The Wnt/beta-catenin pathway regulates cardiac valve formation. Nature 425: 633–637. 14534590 10.1038/nature02028

[bib33] Lage K. Greenway S. C. Rosenfeld J. A. Wakimoto H. Gorham J. M., 2012 Genetic and environmental risk factors in congenital heart disease functionally converge in protein networks driving heart development. Proc. Natl. Acad. Sci. USA 109: 14035–14040. 22904188 10.1073/pnas.1210730109 PMC3435181

[bib34] Larson M. G. Atwood L. D. Benjamin E. J. Cupples L. A. D’agostino R. B. S., 2007 Framingham Heart Study 100K project: genome-wide associations for cardiovascular disease outcomes. BMC Med. Genet. 8(Suppl 1): S5. 17903304 10.1186/1471-2350-8-S1-S5 PMC1995607

[bib35] Lewin M. B. Mcbride K. L. Pignatelli R. Fernbach S. Combes A., 2004 Echocardiographic evaluation of asymptomatic parental and sibling cardiovascular anomalies associated with congenital left ventricular outflow tract lesions. Pediatrics 114: 691–696. 15342840 10.1542/peds.2003-0782-L PMC1361301

[bib36] Li C. Li X. Pang S. Chen W. Qin X., 2014 Novel and functional DNA sequence variants within the GATA6 gene promoter in ventricular septal defects. Int. J. Mol. Sci. 15: 12677–12687. 25036032 10.3390/ijms150712677 PMC4139867

[bib37] Li H. Cherry S. Klinedinst D. Deleon V. Redig J., 2012 Genetic modifiers predisposing to congenital heart disease in the sensitized Down syndrome population. Circ Cardiovasc Genet 5: 301–308. 22523272 10.1161/CIRCGENETICS.111.960872 PMC3386785

[bib38] Li Y. Klena N. T. Gabriel G. C. Liu X. Kim A. J., 2015 Global genetic analysis in mice unveils central role for cilia in congenital heart disease. Nature 521: 520–524. 25807483 10.1038/nature14269 PMC4617540

[bib39] Lignon J. M. Bichler Z. Hivert B. Gannier F. E. Cosnay P., 2008 Altered heart rate control in transgenic mice carrying the KCNJ6 gene of the human chromosome 21. Physiol. Genomics 33: 230–239. 18303085 10.1152/physiolgenomics.00143.2007

[bib40] Lin X. Rinaldo L. Fazly A. F. Xu X., 2007 Depletion of Med10 enhances Wnt and suppresses Nodal signaling during zebrafish embryogenesis. Dev. Biol. 303: 536–548. 17208216 10.1016/j.ydbio.2006.11.034

[bib41] Lin Y. Tseng G. C. Cheong S. Y. Bean L. J. Sherman S. L., 2008 Smarter clustering methods for SNP genotype calling. Bioinformatics 24: 2665–2671. 18826959 10.1093/bioinformatics/btn509 PMC2732271

[bib42] Liu C. Morishima M. Yu T. Matsui S. Zhang L., 2011 Genetic analysis of Down syndrome-associated heart defects in mice. Hum. Genet. 130: 623–632. 21442329 10.1007/s00439-011-0980-2 PMC3257027

[bib43] Liu C. Morishima M. Jiang X. Yu T. Meng K., 2014 Engineered chromosome-based genetic mapping establishes a 3.7 Mb critical genomic region for Down syndrome-associated heart defects in mice. Hum. Genet. 133: 743–753. 24362460 10.1007/s00439-013-1407-z PMC4024075

[bib44] Locke A. E. Dooley K. J. Tinker S. W. Cheong S. Y. Feingold E., 2010 Variation in folate pathway genes contributes to risk of congenital heart defects among individuals with Down syndrome. Genet. Epidemiol. 34: 613–623. 20718043 10.1002/gepi.20518 PMC3378053

[bib45] Ma H. Hao Y. Dong X. Gong Q. Chen J., 2012 Molecular mechanisms and function prediction of long noncoding RNA. ScientificWorldJournal 2012: 541786. 23319885 10.1100/2012/541786 PMC3540756

[bib46] Manning A. L. Ganem N. J. Bakhoum S. F. Wagenbach M. Wordeman L., 2007 The kinesin-13 proteins Kif2a, Kif2b, and Kif2c/MCAK have distinct roles during mitosis in human cells. Mol. Biol. Cell 18: 2970–2979. 17538014 10.1091/mbc.E07-02-0110 PMC1949365

[bib47] Marinou K. Christodoulides C. Antoniades C. Koutsilieris M., 2012 Wnt signaling in cardiovascular physiology. Trends Endocrinol. Metab. 23: 628–636. 22902904 10.1016/j.tem.2012.06.001

[bib48] Martinez S. R. Gay M. S. Zhang L., 2015 Epigenetic mechanisms in heart development and disease. Drug Discov. Today. 20: 799–811. 25572405 10.1016/j.drudis.2014.12.018 PMC4492921

[bib49] Moller J. H. Allen H. D. Clark E. B. Dajani A. S. Golden A., 1993 Report of the task force on children and youth. American Heart Association. Circulation 88: 2479–2486. 8222143 10.1161/01.cir.88.5.2479

[bib50] Pierpont M. E. Basson C. T. Benson D. W. J. Gelb B. D. Giglia T. M., 2007 Genetic basis for congenital heart defects: current knowledge: a scientific statement from the American Heart Association Congenital Cardiac Defects Committee, Council on Cardiovascular Disease in the Young: endorsed by the American Academy of Pediatrics. Circulation 115: 3015–3038. 17519398 10.1161/CIRCULATIONAHA.106.183056

[bib51] Price A. L. Patterson N. J. Plenge R. M. Weinblatt M. E. Shadick N. A., 2006 Principal components analysis corrects for stratification in genome-wide association studies. Nat. Genet. 38: 904–909. 16862161 10.1038/ng1847

[bib52] Pruim R. J. Welch R. P. Sanna S. Teslovich T. M. Chines P. S., 2010 LocusZoom: regional visualization of genome-wide association scan results. Bioinformatics 26: 2336–2337. 20634204 10.1093/bioinformatics/btq419 PMC2935401

[bib53] Puertollano R., 2005 Interactions of TOM1L1 with the multivesicular body sorting machinery. J. Biol. Chem. 280: 9258–9264. 15611048 10.1074/jbc.M412481200

[bib54] Purcell S. Neale B. Todd-Brown K. Thomas L. Ferreira M. A., 2007 PLINK: a tool set for whole-genome association and population-based linkage analyses. Am. J. Hum. Genet. 81: 559–575. 17701901 10.1086/519795 PMC1950838

[bib55] Raid R. Krinka D. Bakhoff L. Abdelwahid E. Jokinen E., 2009 Lack of Gata3 results in conotruncal heart anomalies in mouse. Mech. Dev. 126: 80–89. 18955134 10.1016/j.mod.2008.10.001

[bib56] Ramachandran D. Mulle J. G. Locke A. E. Bean L. J. Rosser T. C., 2015 Contribution of copy-number variation to Down syndrome-associated atrioventricular septal defects. Genet. Med. 17: 554–560. 25341113 10.1038/gim.2014.144 PMC4408203

[bib57] Reller M. D. Strickland M. J. Riehle-Colarusso T. Mahle W. T. Correa A., 2008 Prevalence of congenital heart defects in metropolitan Atlanta, 1998–2005. J. Pediatr. 153: 807–813. 18657826 10.1016/j.jpeds.2008.05.059 PMC2613036

[bib58] Ripoll C. Rivals I. Yahya-Graison E. A. Dauphinot L. Paly E., 2012 Molecular signatures of cardiac defects in down syndrome lymphoblastoid cell lines suggest altered ciliome and hedgehog pathways. PLoS One 7: e41616. 22912673 10.1371/journal.pone.0041616 PMC3415405

[bib59] Robinson S. W. Morris C. D. Goldmuntz E. Reller M. D. Jones M. A., 2003 Missense mutations in CRELD1 are associated with cardiac atrioventricular septal defects. Am. J. Hum. Genet. 72: 1047–1052. 12632326 10.1086/374319 PMC1180336

[bib60] Rosenbloom K. R. Sloan C. A. Malladi V. S. Dreszer T. R. Learned K., 2013 ENCODE data in the UCSC Genome Browser: year 5 update. Nucleic Acids Res. 41: D56–D63. 23193274 10.1093/nar/gks1172 PMC3531152

[bib61] Russo C. A. Elixhauser A., 2007 Healthcare Cost and Utilization Project (HCUP) Statistical Brief #24: Hospitalizations for Birth Defects, 2004. US Agency for Healthcare Research and Quality, Rockville, MD.

[bib62] Sailani M. R. Makrythanasis P. Valsesia A. Santoni F. A. Deutsch S., 2013 The complex SNP and CNV genetic architecture of the increased risk of congenital heart defects in Down syndrome. Genome Res. 23: 1410–1421. 23783273 10.1101/gr.147991.112 PMC3759718

[bib63] Salemi M. Barone C. Romano C. Zolezzi F. Romano C., 2012 Gene expression profiling and qRT-PCR expression of RRP1B, PCNT, KIF21A and ADRB2 in leucocytes of Down’s syndrome subjects. J. Genet. 91: e18–e23. 22552340

[bib64] Sato S. Tomomori-Sato C. Banks C. A. Sorokina I. Parmely T. J., 2003 Identification of mammalian Mediator subunits with similarities to yeast Mediator subunits Srb5, Srb6, Med11, and Rox3. J. Biol. Chem. 278: 15123–15127. 12584197 10.1074/jbc.C300054200

[bib65] Schott J. J. Benson D. W. Basson C. T. Pease W. Silberbach G. M., 1998 Congenital heart disease caused by mutations in the transcription factor NKX2–5. Science 281: 108–111. 9651244 10.1126/science.281.5373.108

[bib66] Shan J. P. Wang X. L. Qiao Y. G. Wan Yan H. X. Huang W. H., 2014 Novel and functional DNA sequence variants within the GATA5 gene promoter in ventricular septal defects. World J. Pediatr. 10: 348–353. 25515806 10.1007/s12519-014-0511-z

[bib67] Shuler C. O. Black G. B. Jerrell J. M., 2013 Population-based treated prevalence of congenital heart disease in a pediatric cohort. Pediatr. Cardiol. 34: 606–611. 22976198 10.1007/s00246-012-0505-3

[bib68] Soemedi R. Wilson I. J. Bentham J. Darlay R. Topf A., 2012 Contribution of global rare copy-number variants to the risk of sporadic congenital heart disease. Am. J. Hum. Genet. 91: 489–501. 22939634 10.1016/j.ajhg.2012.08.003 PMC3511986

[bib69] St Johnston D., 2002 The art and design of genetic screens: *Drosophila melanogaster*. Nat. Rev. Genet. 3: 176–188. 11972155 10.1038/nrg751

[bib70] Tanner S. M. Austin J. L. Leone G. Rush L. J. Plass C., 2001 BAALC, the human member of a novel mammalian neuroectoderm gene lineage, is implicated in hematopoiesis and acute leukemia. Proc. Natl. Acad. Sci. USA 98: 13901–13906. 11707601 10.1073/pnas.241525498 PMC61139

[bib71] Umbhauer M. Djiane A. Goisset C. Penzo-Mendez A. Riou J. F., 2000 The C-terminal cytoplasmic Lys-thr-X-X-X-Trp motif in frizzled receptors mediates Wnt/beta-catenin signalling. EMBO J. 19: 4944–4954. 10990458 10.1093/emboj/19.18.4944 PMC314225

[bib72] Vallaster M. Vallaster C. D. Wu S. M., 2012 Epigenetic mechanisms in cardiac development and disease. Acta Biochim. Biophys. Sin. (Shanghai) 44: 92–102. 22194017 10.1093/abbs/gmr090 PMC3244653

[bib73] Van Rijen E. H. M. Utens E. M. W. J. Roos-Hesselink J. W. Meijboom F. J. Van Domburg R. T., 2005 Longitudinal development of psychopathology in an adult congenital heart disease cohort. Int. J. Cardiol. 99: 315–323. 15749193 10.1016/j.ijcard.2004.09.004

[bib74] Waitzman N. J. Romano P. S. Scheffler R. M., 1994 Estimates of the economic costs of birth defects. Inquiry 31: 188–205. 8021024

[bib75] Wang J. Luo X. J. Xin Y. F. Liu Y. Liu Z. M., 2012 Novel GATA6 mutations associated with congenital ventricular septal defect or tetralogy of Fallot. DNA Cell Biol. 31: 1610–1617. 23020118 10.1089/dna.2012.1814 PMC3482375

[bib76] Wapinski O. Chang H. Y., 2011 Long noncoding RNAs and human disease. Trends Cell Biol. 21: 354–361. 21550244 10.1016/j.tcb.2011.04.001

[bib77] Wilusz J. E. Sunwoo H. Spector D. L., 2009 Long noncoding RNAs: functional surprises from the RNA world. Genes Dev. 23: 1494–1504. 19571179 10.1101/gad.1800909 PMC3152381

[bib78] Xu J. Lin Y. Si L. Jin G. Dai J., 2014 Genetic variants at 10p11 confer risk of tetralogy of Fallot in Chinese of Nanjing. PLoS One 9: e89636. 24594544 10.1371/journal.pone.0089636 PMC3940663

[bib79] Zaidi S. Choi M. Wakimoto H. Ma L. Jiang J., 2013 De novo mutations in histone-modifying genes in congenital heart disease. Nature 498: 220–223. 23665959 10.1038/nature12141 PMC3706629

[bib80] Zheng G. F. Wei D. Zhao H. Zhou N. Yang Y. Q., 2012 A novel GATA6 mutation associated with congenital ventricular septal defect. Int. J. Mol. Med. 29: 1065–1071. 22407241 10.3892/ijmm.2012.930

[bib81] Zwick M. E. Salstrom J. L. Langley C. H., 1999 Genetic variation in rates of nondisjunction: association of two naturally occurring polymorphisms in the chromokinesin nod with increased rates of nondisjunction in *Drosophila melanogaster*. Genetics 152: 1605–1614. 10430586 10.1093/genetics/152.4.1605 PMC1460721

